# A new use for old drugs: identifying compounds with an anti-obesity effect using a high through-put semi-automated *Caenorhabditis elegans* screening platform

**DOI:** 10.1016/j.heliyon.2022.e10108

**Published:** 2022-08-11

**Authors:** Freek Haerkens, Charlotte Kikken, Laurens Kirkels, Monique van Amstel, Willemijn Wouters, Els van Doornmalen, Christof Francke, Samantha Hughes

**Affiliations:** a BioCentre, HAN University of Applied Sciences, 6525EM, Nijmegen, the Netherlands; b Now at the Donders Institute for Brain, Cognition and Behaviour, Radboud University, 6525 AJ, Nijmegen, the Netherlands; c Pivot Park Screening Centre, 5349AB, Oss, the Netherlands; d Now at the Amsterdam Institute for Life and Environment, Environmental Health and Toxicology, Vrije Universiteit Amsterdam, 1081 HV, Amsterdam, the Netherlands

**Keywords:** *C. elegans*, Prestwick chemical library, Obesity, High-throughput screening, Repurposing drugs

## Abstract

Obesity is one of the most common global health problems for all age groups with obese people at risk of a variety of associated health complications. Consequently, there is a need to develop new therapies that lower body fat without the side effects. However, obesity is a complex and systemic disease, so that *in vitro* results are not easily translatable to clinical situations. A promising way to circumnavigate these issues is to reposition already approved drugs for new treatments, enabling a more streamlined drug discovery process due to the availability of pre-existing pharmacological and toxicological datasets. Chemical libraries, such as the Prestwick Chemical Library of 1200 FDA approved drugs, are available for this purpose. We have developed a simple semi-automated whole-organism approach to screening the Prestwick Chemical Library for those compounds which reduce fat content using the model organism *Caenorhabditis elegans*.

Our whole-organism approach to high-throughput screening identified 9 “lead” compounds that reduced fat within 2 weeks in the model. Further screening and analysis provided 4 “hit” compounds (Midodrine, Vinpocetine, Fenoprofen and Lamivudine) that showed significant promise as drugs to reduce fat levels. The effects of these candidates were found to further reduce fat content in nematodes where an *nhr-49*/PPAR mutation resulted in “overweight” worms. Upon unblinding the “hit” compounds, they were found to have recently been shown to have anti-obesity effects in mammalian models too. In developing a whole-animal chemical screen to identify pharmacological agents as potential anti-obesity compounds, we demonstrate how chemical libraries can be rapidly and relatively cheaply profiled for active hits. Using the nematode *Caenorhabditis elegans* thus enables drugs to be assessed for applicability in humans and provides a new incentive to explore drug repurposing as a feasible and efficient way to identify new anti-obesity compounds.

## 1 Introduction

Obesity is defined as having abnormal or excessive fat accumulation in adipose tissue and represents a major public health issue [1]. Obesity is one of the fastest growing health problems in the world [2], with many common diseases such as diabetes and cardiovascular disease are closely linked to being overweight and obese [3]. Current weight loss therapies are lifestyle interventions and bariatric surgery [3, 4], however despite millions of people attempting to lose weight by modifying their diet, this only has a modest result [5]. Consequently, there is growing interest in developing drugs that can promote weight loss and can support battling obesity in combination with diet and exercise therapies [6, 7].

Drugs that promote weight loss is a lucrative market, however the US Food and Drug Administration (FDA) and European Medicines Agency (EMA) have approved just a few [3, 4]. Available since 1999, Orlistat (Alli®, Xenical®) is approved by both the FDA and EMA acting to decrease fat absorption [7, 8]. Many of the other drugs on the market act on the central nervous system, thus indirectly promoting weight loss [3] and the effects of long-term use of these weight-loss drugs remains a concern [8, 9, 10]. Therefore, developing new anti-obesity pharmacological compounds is highly desirable. However, in this drug discovery process, there are two key limiting factors. Firstly, obesity has a complex pathophysiology [3, 4] and secondly, drug development is an expensive, lengthy and high-risk process [11]. To circumnavigate the latter limitation, it is possible to repurpose drugs so that new treatments are quickly bought to the market [12]. Success stories so far include the use of the anti-malaria drug hydroxychloroquine to treat arthritis [13], which has also been suggested to be a suitable anti-obesity drug [14]. In addition, Disulforam, a drug used to treat chronic alcoholism, has been shown to have an anti-obesity effect in mice [15].

The use of the nematode worm *Caenorhabditis elegans* as an alternative screening system to perform a whole-animal chemical screen of drugs is an approach that has been shown to be successful [16, 17, 18]. Indeed, *C. elegans* is ideally placed to identify pharmacological agents that might be potential anti-obesity compounds. Fat distribution and storage pathways are highly conserved between *C. elegans* and mammalian species [19, 20]. Similarly conserved are the pathways for fatty acid biosynthesis, their elongation, and desaturation, mitochondrial and peroxisomal fatty acid β-oxidation, glycolysis, gluconeogenesis and amino acid metabolism [21, 22]. *C. elegans* stores fat as droplets in the intestinal and skin-like epidermal cells, that directly line the intestine. Droplet size, number, and distribution changes dramatically as a response to environmental factors and diet [23].

To visualise the fat content in *C. elegans,* the vital dye Nile Red has been used to stain and quantify fat stores in nematodes [24, 25, 26, 27, 28, 29] without impacting growth, development or survival [30]. Nile Red accumulates in sub-cellular compartments of the nematode intestine, the lysosome-related organelles, which have a strong concentration of polar lipids [27] so that the dye has a strong fluorescence [26, 28, 29, 31], the intensity of which correlates to fat content. This property of Nile Red has allowed it to be used in high throughput screens for molecules and genes that affect fat content and metabolism in *C. elegans* [21, 27, 32, 33, 34, 35, 36, 37, 38]. Indeed Nile Red is extremely useful for high-throughput screening protocols as the dye can be used in a liquid medium [27] so that there is no need for the worms to be fixed, and previous studies have shown a positive correlation between non-fixed Nile Red and expected results [30, 36, 39].

To this end, we developed a platform whereby Nile Red was used in a high-throughput live-stain method to screen the Prestwick Library for compounds that have an effect on fat content in *C. elegans*. The Prestwick Library is a group of 1200 marketed drugs selected for their chemical and pharmacological diversity as well as their known bioavailability and safety (details available at Prestwickchemical.com), so that many of the compounds are FDA-approved drugs. The Prestwick Library is therefore a rich source of potentially bioactive molecules that could be possible anti-obesity treatments.

In our blinded assay, we found 9 compounds that robustly reduced fat content in worms and these were chosen for further analysis. Of these, Midodrine, Vinpocetine, Fenoprofen and Lamivudine are promising pharmacological interventions for treating obesity in humans. This demonstrates that our methodology, using *C. elegans* to screen for fat content in a high throughput manner, is ideally suited in a screen for repurposing compounds for a new role as an anti-obesity drug.

## 2 Results

### 2.1 Assay development

To develop the assay, we chose to use the strain *AW306* (*myo-3::gfp*) which expresses a fluorescent GFP reporter in the body wall muscle which allows for assessment of worm number and normalisation. We tested the number of worms that could be used in the assay to allow a suitable reading from the employed plate reader. Here, a known number of worms were added to wells in a 96-well plate at the L1 stage and grown to L4 in liquid culture before being assessed (Supplemental Figure 1A). We found that the optimal number of worms was 250, as this was a suitable number to grow in the wells without starvation, while the fluorescent value of Nile Red could reach clear and significant levels. Next, we assessed the most suitable concentration of Nile Red using 250 *AW306* worms (Supplemental Figure 1B). These results demonstrated that it was possible to undertake live staining with 0.3μM Nile Red throughout the development of the worms from L1 to L4 stage. This then formed the basis of our obesity assay.

**Figure 1:**
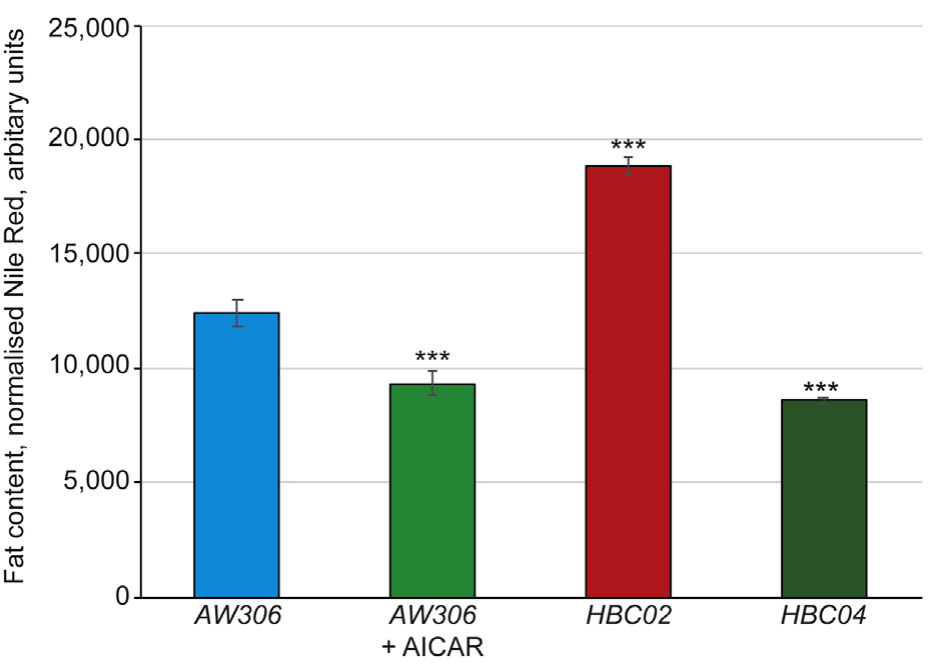
AICAR reduces fat content in a wild type background. Worms (strain, *AW306*) were grown from L1 to L4 in the presence and absence of AICAR in liquid culture in a 96-well plate format. The normalised Nile Red value shows a significant reduction in fat level in AICAR exposed worms (green bar) compared to control (blue bar). Worms which are mutant for *nhr-49* (strain, *HBC02;* red bar) were fatter than controls, while those with a mutation in *cebp-1* (strain, *HBC04*; dark green bar) were thinner. Data is presented as the average ±standard deviation, where *n* = 16 across 4 biologically independent replicates. Asterisks ∗∗∗ indicate a *p* < 0.001 from the 2-tailed 2-sample t-test.

To ensure reproducibility across replicates and to have positive controls, two mutants were used. The gene product of *nhr-49* is a central regulator of fat metabolism [40], and functions in the Peroxisome Proliferator Activated Receptor (PPAR) signalling pathway [21, 41], with mutants for this gene being fat (*STE68*). Another regulator of the fat metabolism is the nematode homolog of mammalian C/EBP, CEBP-1, which when removed from worms makes them thinner than wild type animals (*CZ8920*) [22]. By crossing these strains with *AW306,* which contains the *myo-3::gfp*, to generate *HBC02* and *HBC04*, the resulting population of worms were either fatter or thinner than the wild type *AW306* strain (Figure 1). In addition, the compound AICAR, a compound that is an activator of AMP-activated protein kinase, was included which is known to reduce fat content in worms [36, 42].

To test our assay set-up, we chose to screen 160 randomly chosen compounds from the Prestwick Library of chemicals. We ran the compounds in duplicate on 2 separate 96-well plates. Each plate contained 4 replicates each of the *AW306* strain in the presence and absence of AICAR, *HBC02* (fat) and *HBC04* (thin). After 48 h of incubation, the plates were observed for any obvious changes in worm number, worm development and worm death. All plates had worms that had reached the L4 stage and there was no acute toxicity observed (*data not shown*). The plates were screened resulting in 12 compounds (a hit rate of 7.5%) that reduced fat levels (Table 1). The majority of HTS assays test compounds once or as a duplicate and therefore assays are required that have a high level of accuracy and sensitivity [43]. In general, there is no widely accepted method to evaluate the quality of a HTS, however testing the reproducibility of the controls in each plate by calculating a Z′ factor can provide meaningful data [43]. In the pre-screen of 160 compounds from the Prestwick Library, two Z′ scores were calculated. Firstly, the *AW306* worms in a no compound condition was compared with the same strain in the presence of AICAR, the positive control condition. Here, the Z′ factor was 0.24–0.37 which is classed as a marginal assay. Secondly, the comparison of *AW306* exposed to AICAR with the *HBC02* (the *nhr-49* mutant worms which are fat) resulted in a Z’ factor of 0.59–0.79 suggesting an excellent assay. This gave us the confidence to proceed with the screening of the complete Prestwick Library.

**Table 1:** Result of compounds from the Prestwick library that alter fat content in *C. elegans*. The table shows the compounds that reduced and increased fat content in *C. elegans* for each of the screens of the Prestwick Library of chemicals. The total compounds screened and the percentage of positive hits i.e., those that reduced fat, are shown.

	Relative fat content		Total chemicals screened	Positive hit rate percentage of those compounds that reduce fat
	Reduction	Increase		
Pre-screen	12	0	160	7.5%
Primary Screen	92	52	1200	7.6%
Secondary Screen	29	20	160	18%

### 2.2 Screening of the Prestwick Chemical Library for compounds that reduce fat

The Prestwick Chemical library contains 1200 commercially available pharmacological compounds. These have low toxicity and high biological activity and many have been approved by the US FDA (United States of America Food and Drugs Administration). All compounds were screened in duplicate in a ‘blinded’ fashion at a concentration of 10μM to identify compounds that alter fat content in *C. elegans.* From screening the library, we found that exposure to 92 compounds resulted in a decrease in fat content in the worms, a hit rate of 7.6% (Table 1). These compounds were chosen for a second screen, together with a selection of 26 random compounds that were found to have no effect on fat levels in the first screen and 52 compounds that increased fat from the initial screen. The secondary screen resulted in a positive hit rate of 18%, with 29 compounds identified as causing a decrease in fat content and 20 compounds increasing fat levels in *C. elegans* from both screens (Table 1). The compounds were then un-blinded (please see Supplemental Table 1 for the compounds that robustly reduced fat, and Supplemental Table 2 for those that increased fat content). We chose to focus on the compounds that reduced fat levels in the nematodes for the remainder of this study. Following a literature search, 10 compounds were removed due to their reported anti-helminthic, cytotoxic, antibiotic or contraceptive functions and a further 2 removed as they could not be easily purchased in higher quantities from routine suppliers. The resulting 18 drugs were used in a literature search with 72 different terms (Supplemental Table 3) to identify those that were already known to have an anti-obesity effect. These were not of interest to us, as our purpose was to identify compounds which were likely novel anti-obesity candidates. Ultimately, there were 9 compounds left to investigate in more detail (Supplementary Table 4).

**Figure 2:**
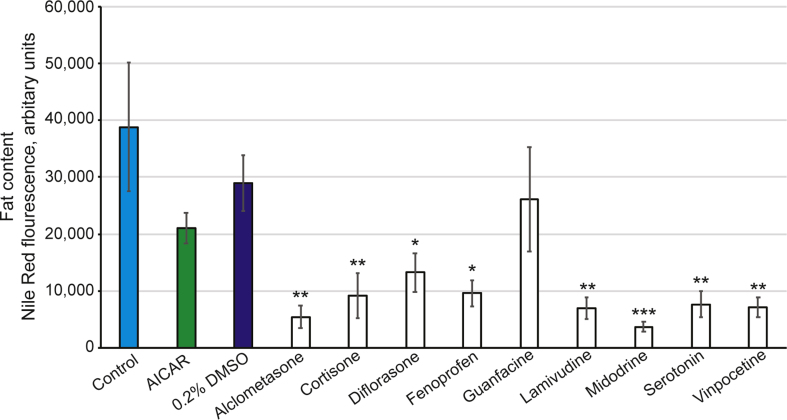
The fat content in worms exposed to drugs is significantly reduced. The 9 lead compounds that gave a positive hit from the chemical library screen, were re-tested in the same manner. All compounds were tested at 10μM using the same liquid assay method and a reduction in fat levels in the worms (strain, *AW306*) was confirmed. All compounds were tested with 4 biological replicates in technical duplicate. The duplicates were combined, the maximal and minimal values, and significant outliers were removed from all samples. The Nile Red fluorescence was normalised to the GFP fluorescence to correct for minor differences in worm number. Data is presented as the average of the normalised Nile Red fluorescence values of the wells with standard error of the mean. Note that the fluorescence values are arbitrary and thus serve as a means to compare fat content. The animals exposed to 0.2% DMSO (vehicle control; dark blue bar) were not significantly different to control animals (blue bar), whereas the AICAR exposed worms (green bar) had a reduction in fat content. Compounds were all tested at 10 μM (white bars) and with the exception of Guanfacine, all show a significant reduction in fat content in populations of animals. The asterisks indicate the *p* value from the 2-tailed 2-sample t-test where ∗*p* < 0.05, ∗∗*p* < 0.025 and ∗∗∗*p* < 0.001.

**Figure 3:**
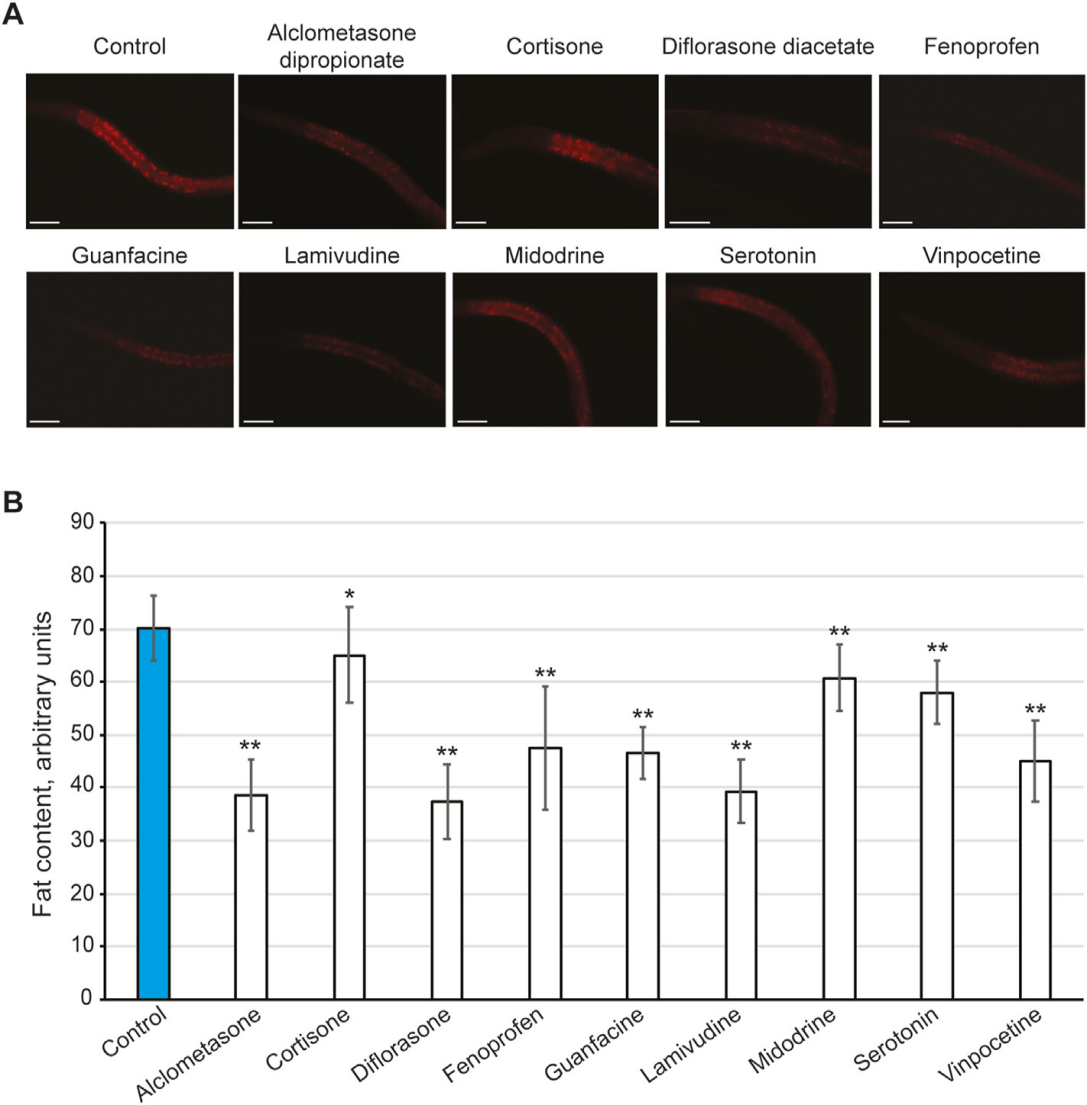
The fat content in worms exposed to drugs on solid media is significantly reduced. The worms were grown from L1 to L4 on solid NGM spiked with 10 μM of each compound. When L4, animals were mounted for microscopy (*n* = 20) and the images exported to ImageJ to quantify the Nile Red intensity as a proxy of fat content (A) Representative pictograms of worms following exposure to the drugs. Images show the anterior of the nematode, all focused on the grinder in the second pharyngeal bulb. All images are taken at the same gain and exposure settings to allow comparison between conditions. Scale bar is 50μm (B) The Nile Red intensity data from ImageJ is presented as the normalised average fat content in animals in arbitrary units with the standard deviation. The statistical analysis results are shown, where ∗ indicates *p* < 0.05 and ∗∗*p* < 0.001.

The worms exposed to the 9 compounds of interest at 10 μM during development from L1 to L4 stage all showed a significant reduction in fat content (Figure 2) and were not found to be toxic to general health parameters (Supplementary Methods and Supplemental Figure 2). To further scrutinise these results, the assay was repeated on solid NGM plates spiked with Nile Red and the compound of interest. Once worms had developed from L1 to L4, several were sacrificed for microscopy to quantify the differences in fat content. As expected, all 9 compounds resulted in a reduction in fat content compared to the controls (Figure 3). Exposure to Midodrine, Serotonin and Cortisone, although significant, was less striking than the reduction induced by the 6 other compounds.

### 2.3 The selected compounds also reduce fat levels in “fat” mutant worms

**Figure 4:**
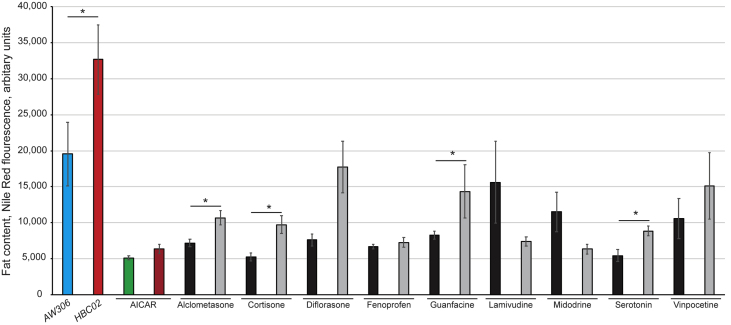
The effect of the compounds of interest in a fat mutant background. Worms were grown from L1 to L4 in liquid culture containing the compound of interest before the fluorescence of Nile Red and GFP assessed in the CLARIOstar® monochromator plate reader. The results were normalised and the data shown is the average with standard error of the mean error bars. The wild type worms (blue bar) and the “fat” worms (strain *HBC02*; red bar) display the relative fat content as expected. The positive control, AICAR, reduced fat in both strains (worms with a wild type background are shown by a green bar and the strain *HBC02* worms exposed to AICAR in dark red). The black bars represent the worms with a wild type genetic background (strain *AW306*) and grey bars are the genetically fat worms (strain *HBC02*). Both strains of worm were exposed to 10 μM compound, with *AW306* as black bars and *HBC02* shown in grey bars. Data is from 2-4 replicates (each with 250 animals) and asterisks show the *p* < 0.05 between indicated samples (2-tailed 2-sample t-test).

The purpose of our pipeline was to identify drugs that are currently on the market that are able to reduce fat and could be repurposed as an anti-obesity drug. To explore how our candidate compounds might be able to alter fat content in an overweight person, a nematode was used with a genetic background that enhanced fat content. The gene product of *nhr-49* is a central regulator of fat metabolism [40], with mutants for this gene being fat. Under control conditions, the strain containing both an *nhr-49* mutation and the *myo-3::gfp* reporter, *HBC02,* was significantly fatter the wild type *AW306* strain. Further, exposure to AICAR resulted in a significant decrease in fat content for both strains to a similar level (Figure 4). Exposure to the compounds of interest resulted in a decrease in fat content in the wild type worms (strain *AW306*), as observed previously. The *nhr-49* mutation (strain, *HBC02*) resulted in all the worms containing much less fat content when exposed to the selected compounds, compared to non-exposed animals. However, in the majority of cases, *HBC02* remained fatter than wild type animals, namely Alclometasone, Cortisone, Diflorasone, Guanfacine, Serotonin and Vinpocetine, suggesting that these drugs may act via the PPAR pathway. Of interest was the effect of Midodrine and Lamivudine on *HBC02*, as these were the only drugs to show a further reduction in fat below that of the control animals and Fenoprofen appeared to reduce fat in both a wild type and *nhr-49* mutant background to a similar level.

## 3 Discussion

Eroom's Law suggests the cost of developing a new drug doubles every 9 years [44, 45]. Currently, it is estimated that to get a drug to market takes up to 15 years and costs 2–3 billion USD per drug [45], while repurposing a drug can take just 6 years at an estimated cost of 300 million USD (45). We show that the use of *C. elegans* in high throughput screens indeed enables compounds to be identified that would normally not be in cell-based *in vitro* screens or would be too expensive or inefficient using an *in vivo* approach. *C. elegans* has been used in this way before, including screening for anti-fungal compounds [46], to locate potential activators of the innate immune system conferring protection against bacterial infection [11] and to identify compounds that activate the Unfolded Protein Response [47].

Our objective was to develop a semi-automated, high throughput screening assay using *C. elegans* to identify compounds that reduce fat content. While it was possible to automate the distribution of the Prestwick Library of 1200 chemicals into 96-well plates for assaying in the worms, for this experiment the worms were distributed into the wells manually. However, we have since been able to dispense worms in an automated fashion, using a Thermo Multidrop Combi, which further reduces the associated manual labour and time required for such screening. There was minimal hands-on time for the screening of the compounds, with most labour dedicated to data analysis. Ultimately, we were successful in screening the full Prestwick Library of FDA approved small molecules in 1 week of practical work in a blinded screen. Further experiments enabled the identification of potential compounds that affect fat content in *C. elegans*, thus supporting our drug repurposing pipeline. It should be noted that only the Prestwick Library was screened and while there may be drugs which are of interest to the field, they may not be present in this library.

Lamivudine was found to reduce fat in a wild type genetic background which is supportive of previous findings that non-nucleoside reverse transcriptase inhibitor drugs, including Lamivudine, have lipoatrophy and pathological fat loss as side-effects [48, 49]. Strikingly, Lamivudine was able to significantly reduce fat in an *nhr-49*/PPAR mutant background, suggesting the effects of this drug will be applicable as an anti-obesity intervention in people with Metabolic Syndrome. Similarly, when Midodrine is administered to rats, the expression of PPAR was upregulated or activated resulting in a lowering of fat and body weight [50, 51]. We also found that worms exposed to Midodrine displayed a reduction in fat content, and in a PPAR mutant background the fat reduction was even more striking. There are some differences between the effects we observed in *C. elegans* and those in mammalian models, which may be due to the fact that the *C. elegans* NHR-49 acts in a similar manner to PPAR-α [52], and the PPAR found to be most responsive to Midodrine in humans was PPAR-γ(50). As Midodrine activates α1-Adrenergic Receptors and has been shown to have few side effects [53], this is a second likely candidate in the arsenal against obesity.

The compounds Fenoprofen and Vinpocetine robustly reduced fat content in all assays. Vinpocetine is a semi-synthetic derivative of the alkaloid vincamine, extracted from the periwinkle plant *Vinca minor*. Vinpocetine has been reported to have minimal side effects at therapeutic doses [54] but has multiple cellular targets including phosphodiesterase-1, voltage gated calcium and sodium channels [55] and IKK [56]. We found that in *C. elegans* Vinpocetine robustly reduced fat content, similar to the effect of the drug in mice [57] and murine cell culture [58] where it was shown to attenuate lipid deposition. Strikingly, Vinpocetine was also one of 60 compounds identified in a screen for enhanced longevity in *C. elegans* [59]. Together, this suggests that Vinpocetine is a highly promising pharmacological intervention to reduce fat content in people who are overweight and obese, and due to the link to the insulin signalling pathway, may confer additional health benefits.

Fenoprofen exposure resulted in a significant reduction in fat content in all worms and genetic backgrounds tested. Fenoprofen activates both peroxisome proliferator activated receptors alpha and gamma (PPAR-α and -γ), to inhibit isozymes of cyclooxygenase COX-2, all of which are linked to obesity [60]. While Fenoprofen has long been used as a nonsteroidal anti-inflammatory drug to treat rheumatoid arthritis, more recently, Fenoprofen was identified as a potential novel therapeutic compound in the treatment of obese patients [61]. Indeed, Fenoprofen has been patented as an anti-obesity drug [62]. Together this strongly places Fenoprofen as a highly likely candidate for repurposing towards a treatment for obesity.

Our goal was to identify compounds with an anti-obesity effect in a simple and efficient manner using the nematode *C. elegans*. As there is a cost associated with developing new drugs, we chose to screen the Prestwick Library of compounds, although this method can be applied to other libraries or compounds individually. One limitation of this study is that false negatives will arise. In any event, this screening methodology identified 4 candidates as anti-obesity compounds, of which many are not yet used as such. Additional studies to examine the mechanism(s) by which the compounds of interest exert their effects on fat metabolism is beyond the scope of this work but will be important to provide indications of how such compounds can be used in the treatment of obesity and applied to humans.

## 4 Conclusion

In conclusion, our *C. elegans* based assay is robust and has the potential to identify novel compounds from a large pool of potential candidates, that can be used to treat obesity. Indeed, our initial high throughput liquid-based assay was undertaken blinded, resulting in 9 lead hit compounds. These were further investigated and four were found to be of significant interest; Midodrine [50], Lamivudine [48], Vinpocetine [58] and Fenoprofen [61] were found to cause weight loss in mammalian models, and we found the same was true of these compounds in *C. elegans*. More strikingly, Fenoprofen, which is a drug currently used to treat rheumatoid arthritis, is now under a patent for its use as an anti-obesity drug [62], and confirmed in our screening pipeline. Together, these data further strengthen the evidence that using our *C. elegans* obesity platform is a quick and efficient method to screen libraries of drugs for their effect on fat content. We have thus developed an assay with which to screen libraries of compounds as a way to repurpose known drugs for new treatments. This contributes to the effort of reducing the extended time taken in the drug discovery pipeline by using a whole-organism approach. Although the use of *C. elegans* is not meant as a replacement for mouse models and clinical trials, it does significantly contribute to the reduction of animal models in testing and shortening the timeframe and costs associated with drug discovery.

## 5 Materials and methods

### 5.1 Strains and maintenance of *Caenorhabditis elegans*

The *C. elegans* strains used in this study were the wild type strains *N2 var.* Bristol, *STE68* [*nhr-49(nr2041*)I] and *CZ8920* [*cebp-1(tm2807*)X], all provided by the *Caenorhabditis* Genetics Centre (CGC, USA). We also utilised *AW306* (*him-8* (*e1489*)V; Is(*myo-3::gfp*))] which was kindly provided by Prof. Alison Woollard (University of Oxford, United Kingdom). *HBC02* [*nhr-49(nr2041*)I; *him-8(e1489*)V; Is(*myo-3::GFP*)] was made by crossing *AW306* with *STE68* with *HBC04* [*cebp-1(tm2807*)X; *him-8(e1489*)V; Is(*myo-3::GFP*)] generated by crossing *AW306* with *CZ8920*. All *C. elegans* strains were maintained on *Escherichia coli OP50* inoculated Nematode Growth Media (NGM) agar following standard protocols [63].

To generate a synchronous population of worms, gravid worms were washed from plates with M9 buffer [63] and bleached using alkaline hypochlorite solution (4 mL 5% sodium hypochlorite, 1 mL 4 M sodium hydroxide, 5 mL water) following standard protocols [64]. Released eggs were left to hatch overnight at room temperature in M9 buffer in the absence of food, so that the synchronised L1 larvae could be used in the assay.

### 5.2 Prestwick Chemical Library screen

**Figure 5:**
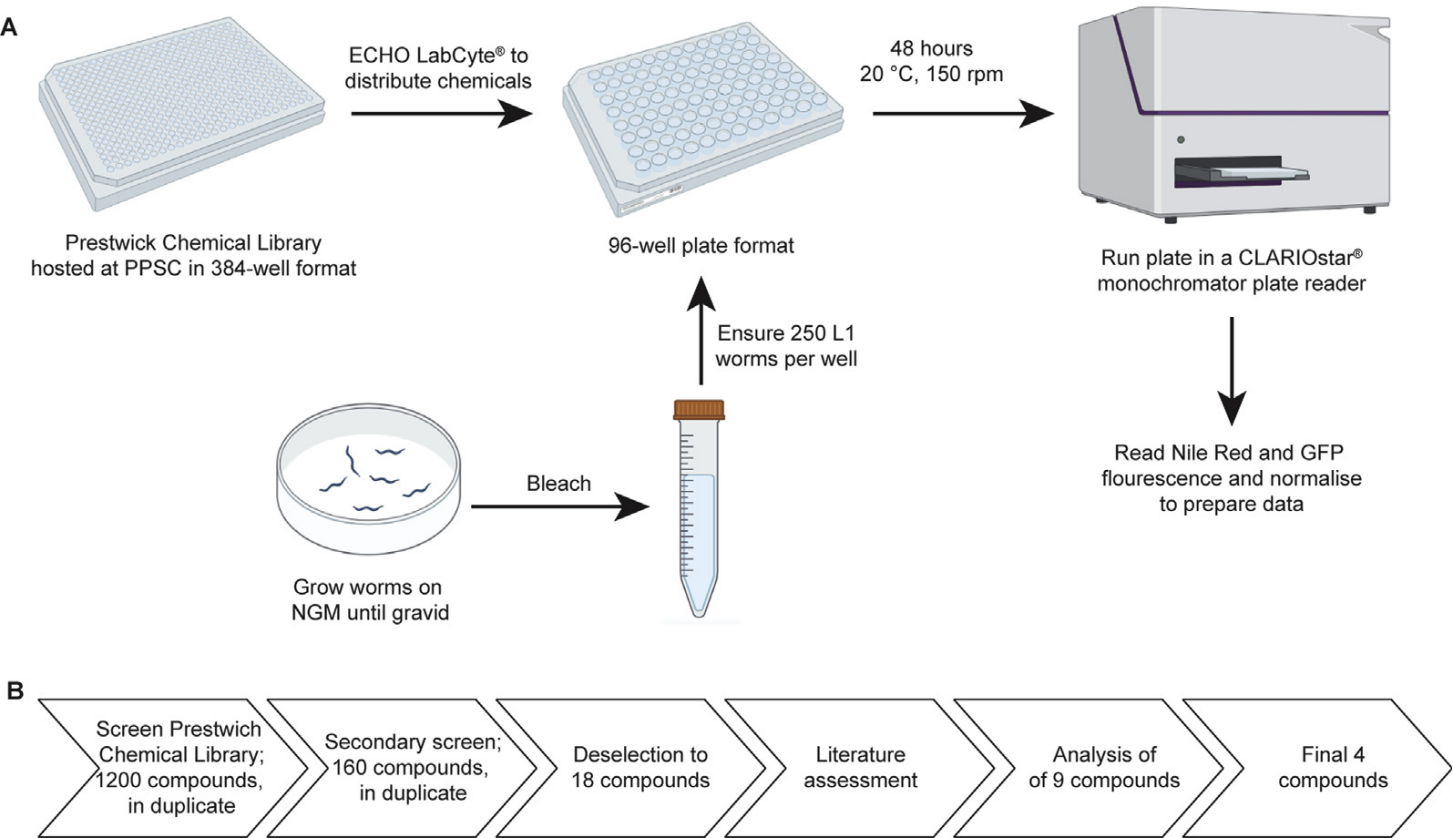
Schematic of the chemical library screen and selection of the final compounds (A) Compounds were transferred from the 384-well plates into 96-well plates for screening by an Echo Labcyte® (Beckman Coulter), both housed at Pivot Park Screening Centre (PPSC). In parallel, *C. elegans* were grown on NGM plates until gravid. Worms were age synchronized and 250 L1 animals added to the 96-well plate. Plates were incubated in a humid chamber at 20 °C, while shaking at 150 rpm for 48 h before being assessed in a CLARIOstar® plate reader (BMG LABTECH) for fluorescence. Figure created with BioRender.com (B) Schematic of how the Prestwick Library of compounds were screened. All 1200 compounds from the Prestwick Library were assessed for an effect on fat content in duplicate. A secondary screen was conducted on 160 compounds, where 52 gave an increase and 92 a decrease in fat levels in *C. elegans* from the initial screen. The second screen resulted in 29 compounds that robustly and significantly reduced fat levels in *C. elegans*. Compounds were deselected based on anti-helminthic, cytotoxic, antibiotic or contraceptive functions and availability. The remaining 18 compounds were subjected to a detailed literature assessment, resulting in a final list of 9 lead compounds (see Table 1).

The Prestwick Chemical Library (www.prestwickchemical.com) comprises 1200 small molecule compounds, where more than 95% of the compounds are marketed drugs (off patent) and the compounds are known to be safe and bioactive in humans. The library was screened using a liquid assay based on a 96-well plate format (see Figure 5 for a schematic of the process). The Pivot Park Screening Centre (PPSC) hosts the Prestwick Library, which is stored at 10 mM concentration in 100% DMSO. Compounds (0.3 μL) were distributed from a 384-well plate format into a 96-well plate (Corning® 96-well black sided clear bottom microplate) format using an Echo Labcyte® (Beckman Coulter, Woerden, the Netherlands), in duplicate. This was done in a “blind” fashion, so it was not known which compound was in which well until the end of the assay. Each well was then filled with 49.7 μL of S-complete [65, 66] containing Nile Red and *OP50 E. coli.* To each well 10 μL M9 buffer containing 250 age-synchronised *AW306* L1 animals was also added, so the wells had a total of 60 μL. The final concentration of the Prestwick Chemical in the well was 10 μM in 0.5% DMSO. Also included on the plates were the control nematode strains (*N2* wild type, *HBC02* and *HBC04*) and 0.1 mM AICAR (5-aminoimiddazole-4-carboxamide 1-ß-D-ribofuranoside, #A611700 from Toronto Research Chemicals, Ontario, Canada) as a positive control compound [36, 42]. The plates were sealed, placed in a shaking humid chamber at 20 °C and 150 rpm. After 48 h, the worms had developed to L4 and the plates were assayed in a CLARIOstar® plate reader (BMG LABTECH, de Meern, the Netherlands).

### 5.3 Drug treatment on solid NGM

Compounds of interest were purchased from Sigma Aldrich and dissolved in DMSO to a stock concentration of 2 mM. To the molten NGM containing Nile Red (final concentration 0.1 μg/mL), compounds were added to a final concentration of 10 μM. The obesity assay on solid NGM was performed as described in van de Klashorst *et al.* [67]. Briefly, Nile Red to a final concentration 0.3 μM was added to molten NGM and the plates were seeded with *OP50 E. coli* when solid. *C. elegans* were added to the plates age synchronised at stage L1 and allowed to develop to stage L4. The L4 worms were washed off the plates with M9 buffer and then pelleted by centrifugation at 2,500 rpm for 90 s. The supernatant was removed and the pellet washed a total of 3 times in M9 buffer. The pellet of worms was resuspended so that there were 250 worms in 60μl M9 buffer which was then placed in a 96-well plate in triplicate and then analysed in the plate reader or selected for microscopy.

### 5.4 Nile red obesity assay

Nile Red (#7726, Roth) was dissolved to 0.5 mg/mL in acetone. The solution was vortexed and left shaking overnight, in the dark. A 1 μg/mL working stock was prepared in 1x PBS and stored at 4 °C in the dark. Prior to starting the experiment, Nile Red was added to S-complete or molten NGM to a final concentration of 0.02 μg/L. Worms at the L1 stage were added to the S-complete (in 10 μL M9 buffer) or directly to the surface of seeded NGM which had been supplemented with Nile Red and the compound of choice. The plates were incubated at 20 °C for 48 h and then analysed using the CLARIOstar®.

The Nile Red fluorescence was read with an excitation wavelength of 528nm and an emission wavelength of 580nm, with GFP fluorescence (a measure of the number of worms based on the *myo-3::gfp* reporter) measured at an excitation and emission wavelength of 482nm and 515nm, respectively. For both spectrums, an 8nm slit was used to ensure a narrow wavelength of light was used to excite the fluorophores. To compare the amount of Nile Red in each well a normalisation calculation was applied, where the presence of the GFP reporter in the body wall muscle from the strain *AW306* was used to normalise to the number of worms. The Nile Red reading was then divided by the normalised GFP value to obtain a normalised Nile Red value.

### 5.5 Microscopy

Worms were mounted on a 2% agarose pad in 0.1% sodium azide and images were taken at x40 magnification on a Zeiss Imager. M2 microscope using Zeiss Zen 2012 Blue software. It should be noted that the fluorescence intensity, gain and exposure settings remained constant for each image and the focal plane was made consistent across worms (by focusing on the grinder in the second pharyngeal bulb). Images were all 2584 × 1936 pixels in size and using ImageJ software v1.51w, the image was converted to an RGB stack. Within ImageJ, a box was drawn with one side touching the grinder. For each image, the box remained the same size (usually 1000 × 1000 pixels). The Nile Red “fluorescence” value was inferred from the Z-axis profile value for the red filter within the drawn box for each worm. Averages were taken of 20 independent animals and these were plotted with the error bars as standard error of the mean. Nile Red “fluorescence” values of the drug exposed animals were compared to the non-exposed control animals using a 2-tailed-2-sample t-test. Representative images were compiled in Adobe Photoshop 7.0.

## Declarations

### Author contribution statement

Freek Haerkens; Laurens Kirkels: Performed the experiments; Analyzed and interpreted the data.

Charlotte Kikken; Willemijn Wouters: Performed the experiments.

Monique van Amstel: Performed the experiments; Contributed reagents, materials, analysis tools or data.

Els van Doornmalen: Conceived and designed the experiments; Contributed reagents, materials, analysis tools or data; Wrote the paper.

Christof Francke: Conceived and designed the experiments; Analyzed and interpreted the data; Contributed reagents, materials, analysis tools or data; Wrote the paper.

Samantha Hughes: Conceived and designed the experiments; Performed the experiments; Analyzed and interpreted the data; Wrote the paper.

### Funding statement

This work was supported by Nationaal Regieorgaan Praktijkgericht Onderzoek SIA [2014-01-07PRO].

Christof Francke was supported by Nationaal Regieorgaan Praktijkgericht Onderzoek SIA [SVB/TOPUP-09-035].

### Data availability statement

Data included in article/supp. material/referenced in article.

### Declaration of interest's statement

The authors declare no conflict of interest.

### Additional information

No additional information is available for this paper.

## Appendix ASupplementary data

Haerkens_SupplementaryMaterial_Heliyon_v3_V2.docx Haerkens_SupplementaryMaterial_Heliyon_v3_V2.docx
