# Supplementary material for: A new use for old drugs: identifying compounds with an anti-obesity effect using a high through-put semi-automated Caenorhabditis elegans screening platform
Source: Heliyon. 2022 Aug 11;8(8):e10108. doi: 10.1016/j.heliyon.2022.e10108 (PMC9399480; doi:10.1016/j.heliyon.2022.e10108)
Supplement: Haerkens_SupplementaryMaterial_Heliyon_v3_V2.docx [file mmc1.docx]

***Supplementary information for:***

**A New Use For Old Drugs: Identifying compounds with an anti-obesity effect using a high through-put semi-automated *Caenorhabditis elegans* screening platform**

Freek Haerkens^1^, Charlotte Kikken^1^, Laurens Kirkels^1,2^, Monique van Amstel^3^, Willemijn Wouters^3^, Els van Doornmalen^3^, Christof Francke^1^, Samantha Hughes^1,4^*

^1^BioCentre, HAN University of Applied Sciences, 6525EM, Nijmegen, the Netherlands

^2^now at the Donders Institute for Brain, Cognition and Behaviour, Radboud University, 6525 AJ, Nijmegen, the Netherlands

^3^Pivot Park Screening Centre, 5349AB, Oss, the Netherlands

^4^now at the Amsterdam Institute for Life and Environment, Environmental Health and Toxicology, Vrije Universiteit Amsterdam, 1081 HV, Amsterdam, the Netherlands

*** Corresponding author:**

Samantha Hughes: s.hughes@vu.nl

**This file includes:**

Supplementary Methods

Supplementary Figure 1: Development and optimisation of high throughput screening assay.

Supplementary Figure 2: The effect of the hit compounds on reproduction and development of *C. elegans.*

Supplemental Table 1: List of 29 fat-reducing hits.

Supplemental Table 2: List of 20 hits that increase fat content.

Supplemental Table 3: List of the 72 search terms used in the literature search.

Supplemental Table 4: List of final 9 potential anti-obesity drugs

# Supplementary Methods

*1.1 Development and reproduction assay*

A simple development and reproductive toxicity (DART) assay was performed to assess the toxicity of the compounds in S-complete liquid culture. A single L4-stage wild type nematode in M9 buffer was added to each well of a 96-wells plate containing S-complete, bacteria and the compound of interest at 10µM, with an *n*<12. Two independent biological replicates were undertaken and the data combined. The plates were incubated for 48 hours at 20^o^C with gentle shaking at 150 rpm. Each well was assessed for the brood size and development of the progeny. An estimate of the brood size was recorded in three groups: where “0” indicated that there was no offspring produced’ “1” indicated up to 10 viable offspring; “2” was a score for 11-75 viable progeny; “3” represented a wild type bin where there was more than 75 viable progeny. In addition, the mobility of the offspring was observed and also classified into three groups: “2-Healthy’, where progeny showed normal development and head-to-tail body bending; “1-impaired development’, where there was a developmental delay and more than 50% of the progeny had defective mobility; “3-Dead or not moving’ was scored where the majority of the progeny were dead or not moving.

# Supplementary Figures


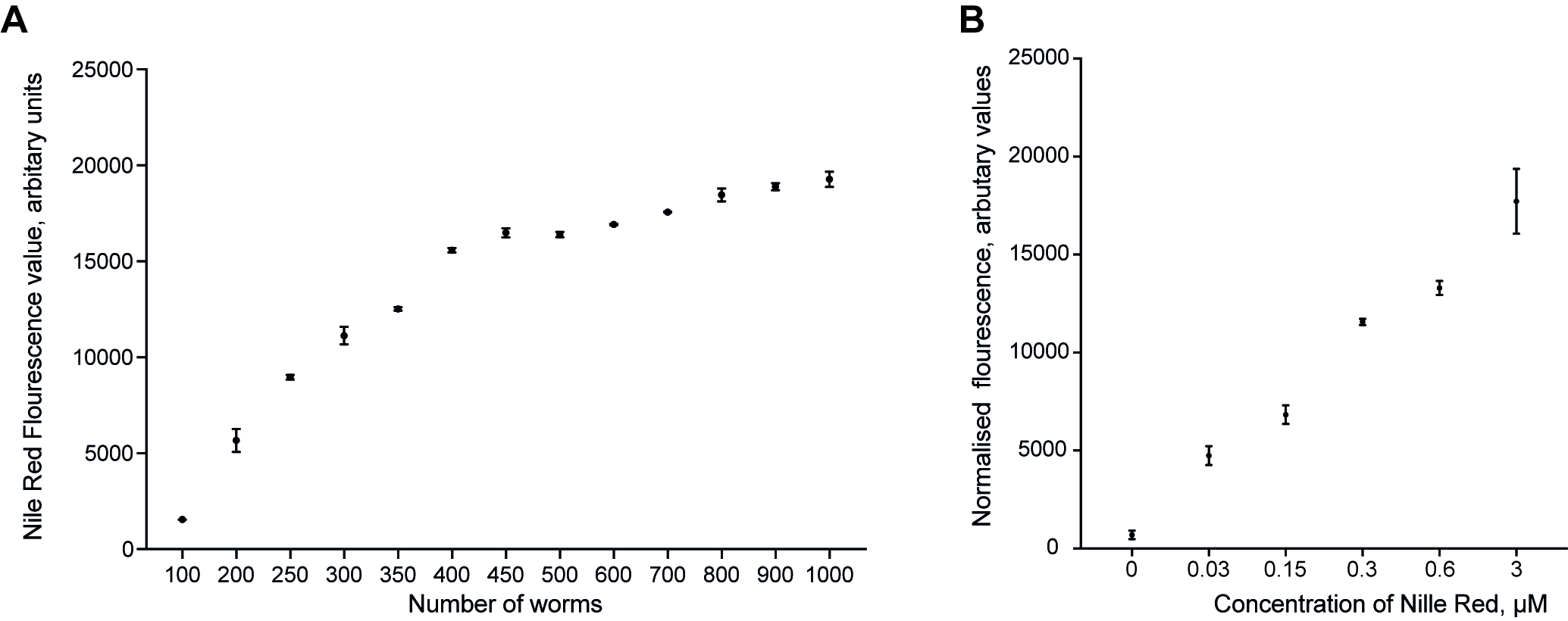


**Supplemental figure 1. Development and optimisation of high throughput screening assay. (A)** Assessment of number of worms to be used in the assay. Following the age synchronisation of *C. elegans*, the number of L1 was counted and the number indicated was added to wells of a 96-well plate in triplicate. The plates were incubated at 20^o^C for 48 hours with gentle shaking (150 rpm) to allow the worms to develop to L4. The amount of Nile Red fluorescence was assessed at least 2 independent biological replicates. The results were plotted with the standard deviation. **(B)** Identification of suitable Nile Red concentration. Worms (250 at L1 stage) were exposed to increasing concentrations of Nile Red for 48 hours before being assessed. The fluorescent values were calculated and normalised (as described in the Materials and Methods section). The data from 4 replicates was averaged and plotted with the standard deviation.


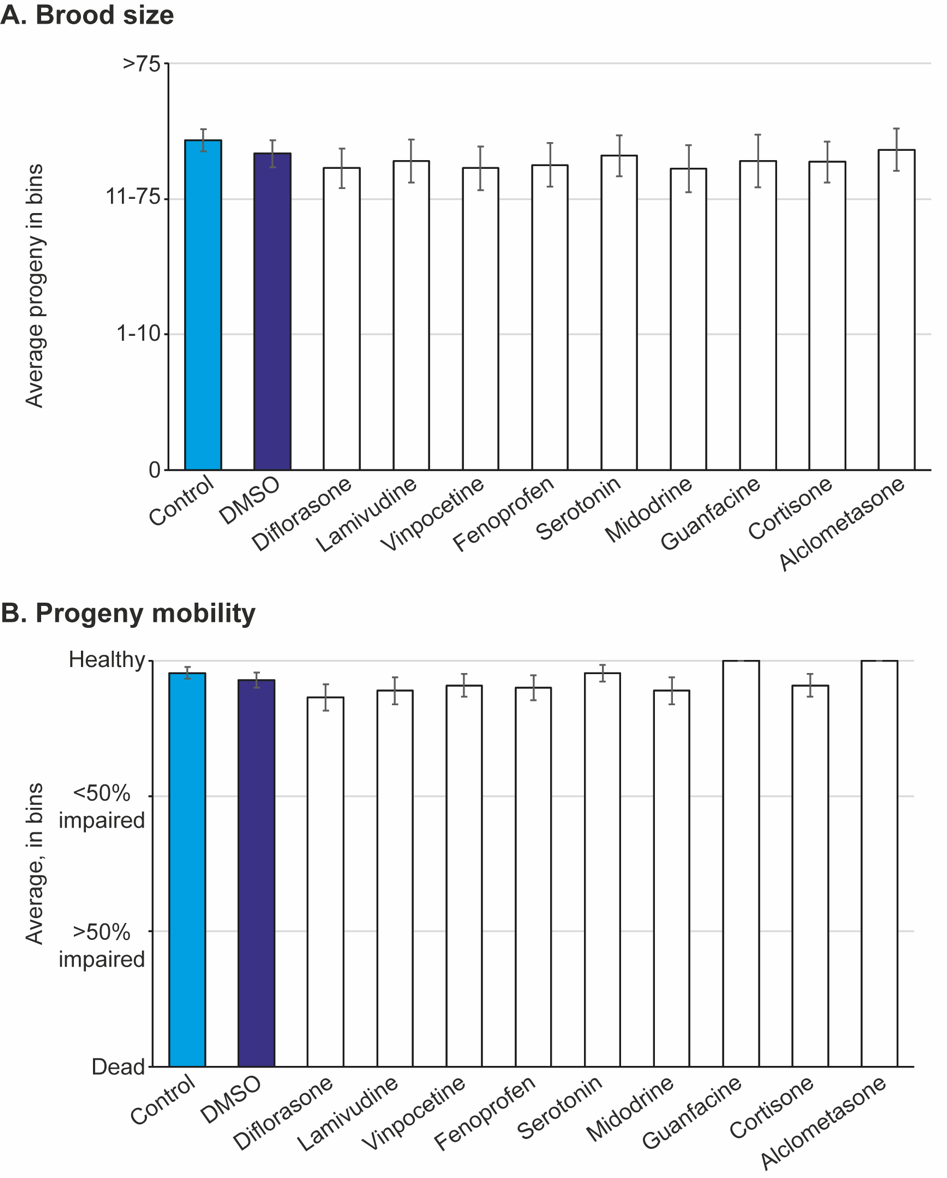


**Supplementary Figure 2.** **The effect of the hit compounds on reproduction and development of *C. elegans*.** Each compound was screened at a concentration of 10µM in 0.2% DMSO. A single L4 worm was added to each well and incubated at 20^o^C with gentle agitation (at 150 rpm). After 48 hours, each well was assessed for the brood size and mobility of the progeny. Data is presented as the average ± standard error of the mean, across 2 biologically independent replicates. **(A)** Brood size of worms exposed to the compounds after 48 hours was classified into “0” (no viable offspring), “1” (1-10 viable offspring), “2” (11-75 viable offspring) and “3” more than 75 viable offspring. **(B)** Mobility of the offspring was classified into “healthy”, “impaired” or “dead”.

**Supplemental Table 1: List of 29 fat-reducing hits.** After the second screen, the 29 compounds that robustly and significantly reduced fat levels across all screens were de-blinded. From these compounds, 11 were removed because they were anti-helminthic, cytotoxic, were an antibiotic or could not be purchased. The remaining compounds were selected for a literature search and their main biological

| **Drug name** | **Biological effect** | **Reason for deselection** |
| --- | --- | --- |
| Aceclidine Hydrochloride | treatment for glaucoma |  |
| Acetaminophen | n/a | antibacterial |
| Adrenosterone | steroid hormone |  |
| Alclometasone dipropionate | glucocorticoid corticosteroid |  |
| Bemegride | respiratory stimulant |  |
| Bezafibrate | n/a | cytostatic |
| Bisoprolol fumarate | n/a | cytostatic |
| Chlorphensin carbamate | n/a | Could not purchase |
| Cortisone | glucocorticoid corticosteroid |  |
| Diflorasone Diacetate | anti-inflammatory |  |
| Dosulepin hydrochloride | antidepressant |  |
| Fendiline hydrochloride | n/a | cytostatic |
| Fenoprofen calcium salt dihydrate | NSAID for inflammation and arthritis |  |
| Flecainide acetate | anti-arrhythmia |  |
| Fluconazole | anti-fungal |  |
| Guanfacine hydrochloride | ADHD and high blood pressure |  |
| (+)-Isoproterenol (+)-bitartrate salt | hypertension and angina pectoris |  |
| Lamivudine | HIV/AIDS and hepatitis B |  |
| Midodrine hydrochloride | vasoconstrictor and antihypertension |  |
| Modafinil | narcolepsy and as a cognitive enhancer | Could not purchase |
| Moxifloxacin | n/a | antibacterial |
| Opipramol dihydrochloride | antidepressant |  |
| Parbendazole | n/a | anti-helminth |
| Pioglitazone | n/a | antibacterial |
| Pindolol | hypertension and angina pectoris |  |
| Serotonin hydrochloride | antidepressant |  |
| Sulfanilamide | n/a | antibacterial |
| Sulfaguanidine | n/a | antibacterial |
| Vinpocetine | anti-inflammatory |  |

**Supplemental Table 2: List of 20 hits that increase fat content.** The first screen identified 52 compounds that increased fat. After the second screen, the 20 compounds that robustly and significantly increased fat levels across both screens were de-blinded.

| Bextaxolol hydrochloride | Meclozine dihydrochloride | Probucol |
| --- | --- | --- |
| Busulfan | Mepivacaince hydrochloride | Quetiapine hemifumerate |
| Cilnidipine | Meropenem | Sipierone |
| Dehydrocholic acid | Oxcarbazepine | Terfenadine |
| Dihydrostrepromycin sulfate | Oxfendazol | Tetracycline hydrochloride |
| Flubendazol | Oxybutynin chloride | Toreifene |
| Homochlorcyclizine dihydrochloride | Oxytetracycline dihydrate |  |

**Supplemental Table 3: List of the 72 search terms used in the literature search.** After the 2 rounds of screening, the positive hits were un-blinded (Supplemental Table 1) and subjected to a literature search using specific fat or obesity related terms. Compounds with a known anti-obesity effect were removed from the hit-list.

| Adipose tissue | Diet | LDL | Polyunsaturated fatty acids |
| --- | --- | --- | --- |
| Anorexic effects | Dose | Lethal concentration | Progeny |
| Anti-obesity | Dose response | Lethal dose | Rats |
| Appetite | Eating | Lethality | Reproduction |
| Appetite depressant | Eggs | Lifespan | Rodents |
| Appetite suppressant | Fat | Lipids | Skeletal |
| Blood pressure | Fat distribution | Low density lipids | Stress |
| Bodipy | Fat redistribution | Mammals | Sudan Black |
| Bullemia | Fatty acids | Metabolism | Sugar |
| *C. elegans* | Fitness | Mice | Survival |
| *Caenorhabditis elegans* | HDL | Model systems | TAG |
| Calorific restriction | Health | Muscle | Triacylglycerides |
| Cholesterol | Heart disease | Nile red | Unsaturated fatty acids |
| Clinical trial | High density lipids | Obesity | Vertebrates |
| Death | Immune response | Offspring | Vital dyes |
| Depression anorexia | Invertebrates | Oil red O | Weight gain |
| Diabetes | LC50 | Pain/analgesia | Weight loss |
| Diarrhoea | LD50 | Pharyngeal pumping | Worms |

**Supplemental Table 4. List of final 9 potential anti-obesity drugs.** The compound is given with its chemical formula and molecular weight (g/mol) together with the known effect of the compound. Information was obtained from Pubchem or references as shown. The supplier of these compounds is also shown.

| **Compound** | **Function** | **Chemical formula and Molecular weight** | **Supplier** |
| --- | --- | --- | --- |
| Alclometasone dipropionate | A synthetic glucocorticoid receptor agonist mimicking the metabolic, anti-inflammatory, immunosuppressive, antipruritic, and vasoconstrictive effects of natural glucocorticoids. Used to treat glaucoma(67). | C_28_H_37_ClO_7_, 521g/mol | Sigma Aldrich (#1012757) |
| Cortisone | A glucocorticoid receptor antagonist used to treat allergies or inflammation(68). | C_21_H_28_O_5_, 360.4g/mol | Sigma Aldrich (#C2755) |
| Diflorasone diacetate | A synthetic glucocorticoid with anti-inflammatory and immunosuppressive properties, commonly used as a skin treatment. | C_26_H_32_F_2_O_7_, 494.5g/mol | Sigma Aldrich (#1197302) |
| Fenoprofen | A derivative of propionic acid with analgesic, non-steroidal anti-inflammatory and anti-rheumatic properties. Fenoprofen inhibits isozymes of cyclooxygenase COX-2(64). | C_15_H_14_O, 242.2699g/mol | Sigma Aldrich (#F1517) |
| Guanfacine | An alpha-2 adrenergic agonist with non-stimulant and antihypertensive activity. Used to treat ADHD and high blood pressure(69). | C_9_H_9_C_l2_N_3_O, 246.093g/mol | Sigma Aldrich (#PHR1751) |
| Lamivudine | A synthetic nucleoside analogue that is used against hepatitis B virus and HIV infection. Lamivudine is phosphorylated to its active metabolites of which Lamivudine triphosphate (L-TP) is a weak inhibitor of DNA polymerases and mitochondrial DNA polymerase(70). | C_8_H_11_N_3_O_3_S, 229.26g/mol | Sigma Aldrich (#L1295) |
| Midodrine | A direct acting pro-drug that is a sympathomimetic agent with anti-hypotensive properties. Midodrine is converted to its active metabolite, desglymidodrine, which selectively binds to and activates alpha-1-adrenergic receptors of the arteriolar and venous vasculature(71). | C_12_H_18_N_2_O_4_, 254.285g/mol | Sigma Aldrich (#M8277) |
| Serotonin | A biochemical messenger and neurotransmitter, otherwise known as 5-hydroxytryptamine (5-HT). | C_10_H_12_N_2_O, 176.21g/mol | Sigma Aldrich (#H9523) |
| Vinpocetine | An alkaloid that inhibits inflammatory responses through the TLR4/MyD88/NF-κB signalling pathway. Used as a cognitive enhancement “memory booster” and as an anti-inflammatory(72). | C_22_H_26_N_2_O_2_, 350.454g/mol | Sigma Aldrich (#V6383) |
